# Supplementary material for: LinguaPhylo: A probabilistic model specification language for reproducible phylogenetic analyses
Source: PLoS Comput Biol. 2023 Jul 18;19(7):e1011226. doi: 10.1371/journal.pcbi.1011226 (PMC10381047; doi:10.1371/journal.pcbi.1011226)
Supplement: S1 Appendix — (PDF) [file pcbi.1011226.s001.pdf]

Supplementary Material for "LinguaPhylo: a probabilistic model specification language for reproducible phylogenetic analyses"

Alexei J. Drummond[1,2,3]*, Kylie Chen[1,2,3], Fábio K. Mendes[1,4], Dong Xie[1,2,3]

1 Centre for Computational Evolution, University of Auckland, Auckland, New Zealand

2 School of Biological Sciences, University of Auckland, Auckland, New Zealand

3 School of Computer Science, University of Auckland, Auckland, New Zealand

4 Department of Biology, Washington University in St. Louis, St. Louis, United States

*Corresponding author: a.drummond@auckland.ac.nz

List of Tables

A Substitution models and rate matrix functions. 3
B Coalescent tree generative distributions. 3
C Birth-death tree generative distributions. 4
D Phylogenetic likelihood distributions. 4
E Parametric distributions. 5
F Alignment data types. 6
G Bayesian phylogenetic site model averaging. 6

Function	Description	Examples
binaryRateMatrix	Binary trait rate matrix	errorModel1.lphy, errorModel2.lphy
f81	F81 model[1]	f81Coalescent.lphy
generalTimeReversible	General time reversible rate matrix	h5n1.lphy
gtr	GTR model[2]	gtrCoalescent.lphy
hky	HKY model[3]	hkyCoalescent.lphy
jukesCantor	Jukes-Cantor model[4]	jcCoalescent.lphy
k80	K80 model[5]	
lewisMK	LewisMK model[6]	lewisMKCoalescent.lphy
migrationMatrix	Population process rate matrix	simpleStructuredCoalescent.lphy
wag	WAG model[7]	wagCoalescent.lphy

Table A: Substitution models and rate matrix functions.

Generative distribution	Description	Examples
MultispeciesCoalescent	Multispecies coalescent	simpleMultispeciesCoalescent.lphy, simpleMultispeciesCoalescentTaxa.lphy, twoGeneMultispeciesCoalescent.lphy
Coalescent	Kingman's coalescent [8]	RSV2.lphy
SkylineCoalescent	Skyline coalescent [9]	hcv_col.lphy
StructuredCoalescent	Structured coalescent[10]	simpleStructuredCoalescent.lphy

Table B: Coalescent tree generative distributions.

Generative distribution	Description	Examples
BirthDeathSampling	Birth-death-sampling tree[11, 12]	birthDeathRhoSampling.lphy
BirthDeathSerialSampling	Birth-death serial sampling tree[13]	simpleBirthDeathSerial.lphy
BirthDeath	Calibrated birth-death[14]	simpleCalibratedBirthDeath.lphy, simpleExtantBirthDeath.lphy
FossilBirthDeathTree	Fossilized birth-death process[15]	simFossilsCompact.lphy
FullBirthDeath	Birth-death tree[16]	simpleFullBirthDeath.lphy
RhoSampleTree	Birth-death tree sampled from a larger tree	
SimBDReverse	Birth-death tree with extant and extinct species	simFossils.lphy
SimFBDAge	Birth-death tree with extant and extinct species sampled through time	simFBDAge.lphy
SimFossilsPoisson	Tree with fossils added to given tree at rate psi	simFossils.lphy
Yule	Yule tree[17]	simpleYule.lphy, yuleRelaxed.lphy

Table C: Birth-death tree generative distributions.

Generative distribution	Description	Examples
PhyloBrownian	Brownian motion process[18]	simplePhyloOU.lphy
PhyloCTMC	Continuous time Markov process[1]	simpleBModelTest.lphy
PhyloMultivariateBrownian	Multivariate Brownian motion	simplePhyloMultivariateBrownian.lphy
PhyloOU	Ornstein-Ulhenbeck process[18]	simplePhyloBrownian.lphy

Table D: Phylogenetic likelihood distributions.

Generative distribution	Description	Examples
Bernoulli	Bernoulli distribution	simpleRandomLocalClock.lphy, simpleBModelTest.lphy
Beta	Beta distribution	birthDeathRhoSampling.lphy, simpleBModelTest.lphy
Cauchy	Cauchy distribution	
Dirichlet	Dirichlet distribution	birthDeathRhoSampling.lphy, dirichlet.lphy
DiscreteUniform	Discrete-uniform distribution	simpleBModelTest.lphy, simpleBModelTest2.lphy
DiscretizeGamma	Discretize-gamma distribution	gtrGammaCoalescent.lphy, simpleBModelTest.lphy
Exp	Exponential distribution	birthDeathRhoSampling.lphy, yuleRelaxed.lphy
ExpMarkovChain	Smoothing distribution [9]	skylineCoalescent.lphy
Gamma	Gamma distribution	covidDPG.lphy
Geometric	Geometric distribution	
InverseGamma	Inverse-gamma distribution	totalEvidence.lphy
LogNormal	Log-normal distribution	hkyCoalescent.lphy, errorModel1.lphy
Normal	Normal distribution	simplePhyloBrownian.lphy, simplePhyloOU.lphy
NormalGamma	Normal-gamma distribution	simplePhyloBrownian.lphy, simplePhyloOU.lphy
Poisson	Poisson distribution	expression4.lphy, simpleRandomLocalClock2.lphy
RandomBooleanArray	Samples a random boolean array	simpleRandomLocalClock2.lphy
RandomComposition	Samples a random k-tuple of positive integers that sum to n	skylineCoalescent.lphy
Uniform	Uniform distribution	simFossilsCompact.lphy
Weibull	Weibull distribution	
WeightedDirichlet	Weighted dirichlet distribution	totalEvidence.lphy, weightedDirichlet.lphy

Table E: Parametric distributions.

Function	Description	Examples
aminoAcids	Amino acid data type	wagCoalescent.lphy
binaryDataType	Binary data type	
nucleotides	Nucleotide data type	primates2.lphy
standard	Standard data type	totalEvidence.lphy

Table F: Alignment data types.

Function	Description	Examples
nucleotideModel	bModelTest[19] rate matrix	simpleBModelTest.lphy, simpleBModelTest2.lphy
bModelSet	bModelTest model set	simpleBModelTest.lphy
bSiteRates	Site rates for the given bModelTest parameters	simpleBModelTest2.lphy
bSiteModel	bModelTest site model	simpleBModelTest.lphy

Table G: Bayesian phylogenetic site model averaging.

References

[1] Felsenstein J. Evolutionary trees from DNA sequences: a maximum likelihood approach. J Mol Evol. 1981;17(6):368–76. doi:10.1007/BF01734359.

[2] Tarvaré S. Some probabilistic and statistical problems in the analysis of DNA sequences. Some mathematical question in biology-DNA sequence analysis. 1986;.

[3] Hasegawa M, Kishino H, Yano T. Dating of the human-ape splitting by a molecular clock of mitochondrial DNA. Journal of molecular evolution. 1985;22(2):160–174.

[4] Jukes TH, Cantor CR, et al. Evolution of protein molecules. Mammalian protein metabolism. 1969;3:21–132.

[5] Kimura M. A simple method for estimating evolutionary rates of base substitutions through comparative studies of nucleotide sequences. Journal of molecular evolution. 1980;16(2):111–120.

[6] Lewis PO. A likelihood approach to estimating phylogeny from discrete morphological character data. Systematic biology. 2001;50(6):913–925.

[7] Whelan S, Goldman N. A general empirical model of protein evolution derived from multiple protein families using a maximum-likelihood approach. Molecular biology and evolution. 2001;18(5):691–699.

[8] Rodrigo AG, Felsenstein J. Coalescent Approaches to HIV Population Genetics. In: K C, editor. The Evolution of HIV. Baltimore: Johns Hopkins Univ. Press; 1999.

[9] Drummond A, Rambaut A, Shapiro B, Pybus O. Bayesian coalescent inference of past population dynamics from molecular sequences. Molecular biology and evolution. 2005;22:1185–1192. doi:10.1093/molbev/msi103.

[10] Müller NF, Rasmussen DA, Stadler T. The structured coalescent and its approximations. Molecular biology and evolution. 2017;34(11):2970–2981.

[11] Stadler T. Mammalian phylogeny reveals recent diversification rate shifts. Proceedings of the National Academy of Sciences. 2011;108(15):6187–6192.

[12] Stadler T, Kouyos R, von Wyl V, Yerly S, Böni J, Bürgisser P, et al. Estimating the Basic Reproductive Number from Viral Sequence Data. Molecular biology and evolution. 2012;29:347–357. doi:10.1093/molbev/msr217.

[13] Stadler T, Yang Z. Dating phylogenies with sequentially sampled tips. Syst Biol. 2013;62(5):674–88. doi:10.1093/sysbio/syt030.

[14] Heled J, Drummond AJ. Calibrated birth-death phylogenetic time-tree priors for Bayesian inference. Systematic Biology. 2015;64(3):369–383.

[15] Heath TA, Huelsenbeck JP, Stadler T. The fossilized birth-death process for coherent calibration of divergence-time estimates. Proceedings of the National Academy of Sciences. 2014;111(29):E2957–E2966.

[16] Kendall DG. On the generalized" birth-and-death" process. The annals of mathematical statistics. 1948;19(1):1–15.

[17] Yule GU. A mathematical theory of evolution, based on the conclusions of Dr. JC Willis, FRS. Philosophical Transactions of the Royal Society of London Series B. 1925;213:21–87.

[18] Felsenstein J. Maximum-likelihood estimation of evolutionary trees from continuous characters. American journal of human genetics. 1973;25(5):471.

[19] Bouckaert R, Drummond A. bModelTest: Bayesian phylogenetic site model averaging and model comparison. BMC Evolutionary Biology. 2017;17(42). doi:10.1186/s12862-017-0890-6.
