# Supplementary material for: LinguaPhylo: A probabilistic model specification language for reproducible phylogenetic analyses
Source: PLoS Comput Biol. 2023 Jul 18;19(7):e1011226. doi: 10.1371/journal.pcbi.1011226 (PMC10381047; doi:10.1371/journal.pcbi.1011226)

# Dependencies

- Parameter changes are propagated down the workflow

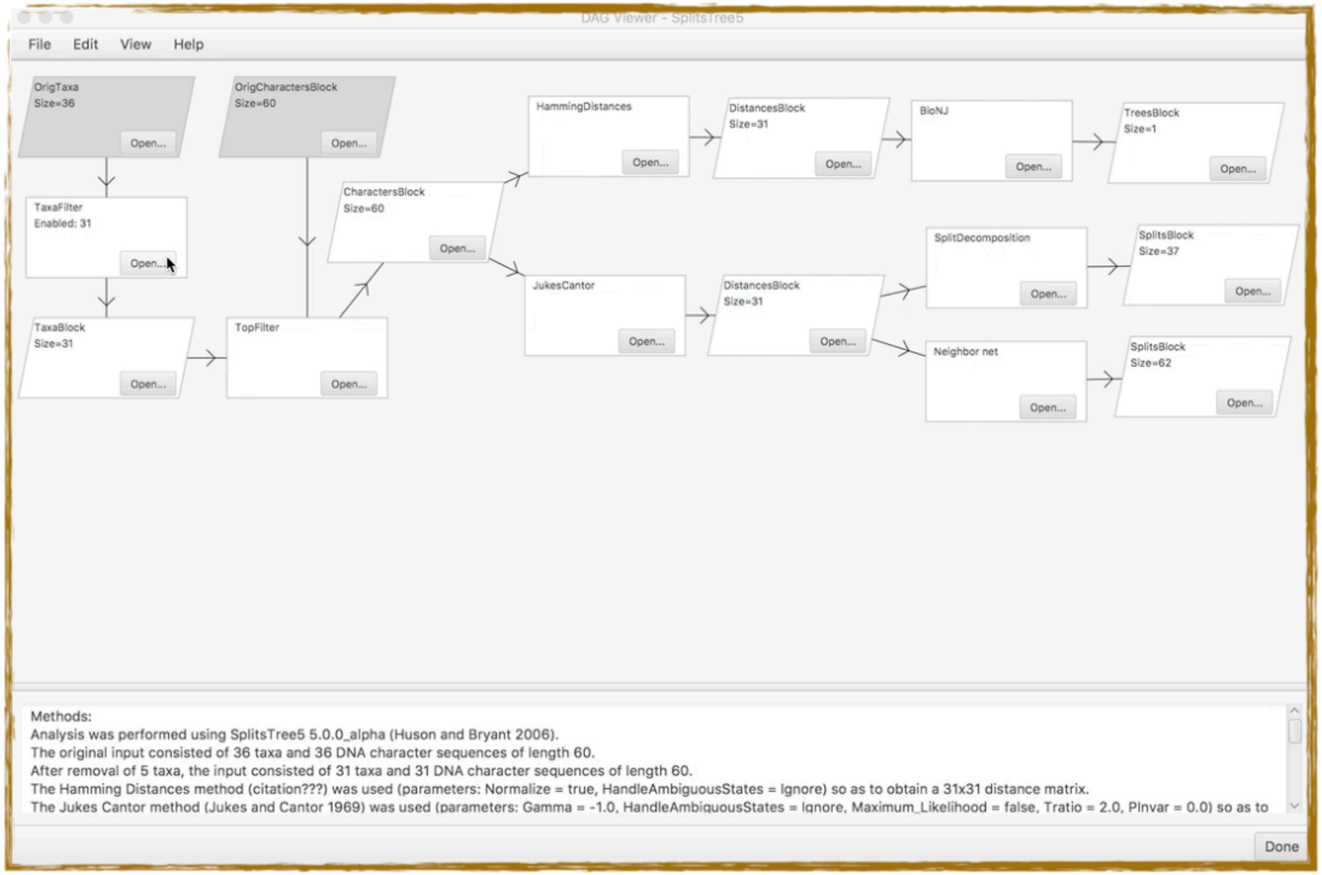

EBERHARD KARLS
UNIVERSITÄT
TÜBINGEN
Center for Bioinformatics

# Methods Section Generator

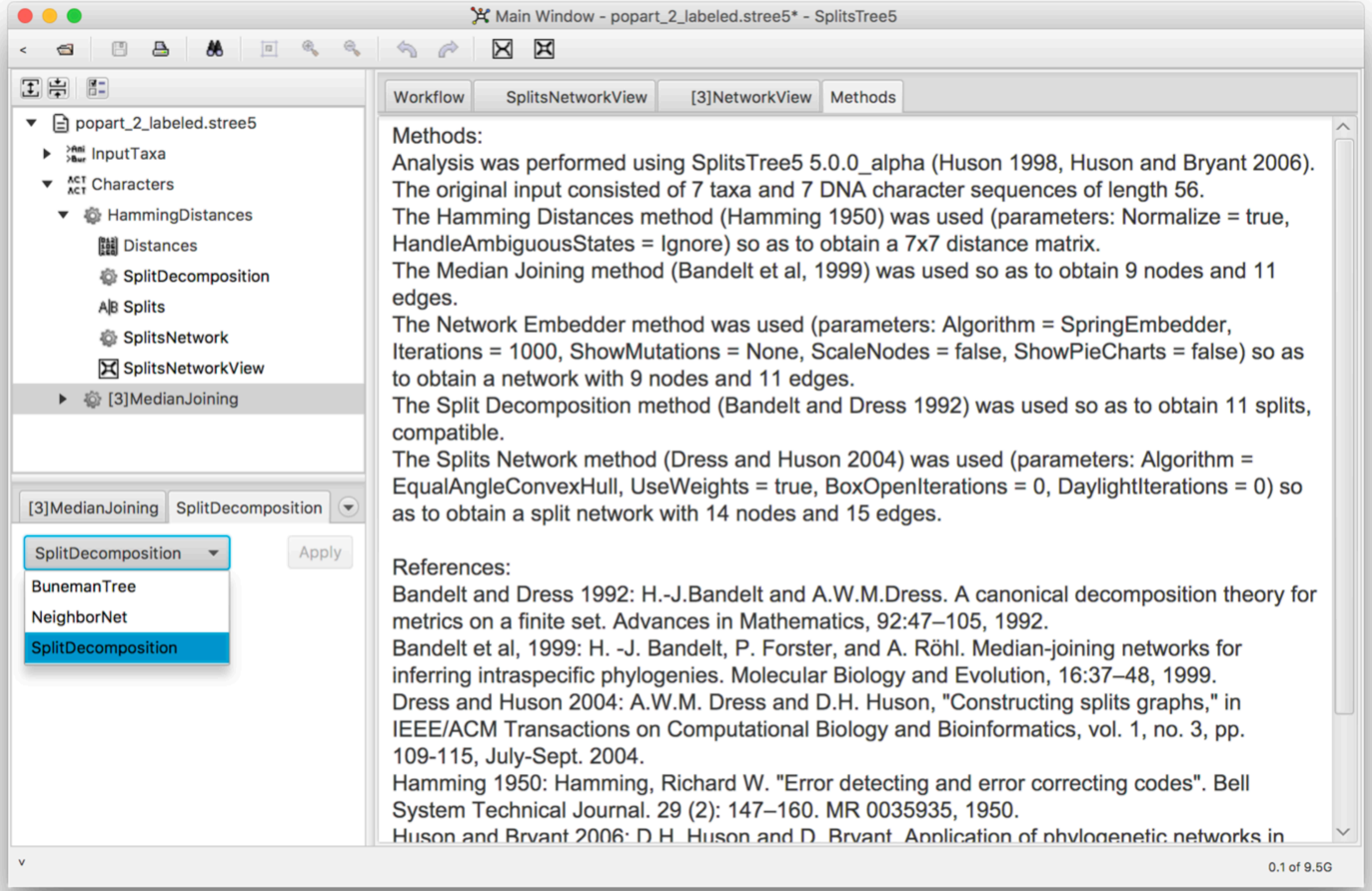

Supplement: Attachment — Submitted filename: Huson-2018.pdf [file pcbi.1011226.s002.pdf]
