# Supplementary material for: LinguaPhylo: A probabilistic model specification language for reproducible phylogenetic analyses
Source: PLoS Comput Biol. 2023 Jul 18;19(7):e1011226. doi: 10.1371/journal.pcbi.1011226 (PMC10381047; doi:10.1371/journal.pcbi.1011226)
Supplement: Attachment — Submitted filename: response-to-reviewers.docx [file pcbi.1011226.s003.docx]

### Response

We thank the editors and reviewers for their valuable feedback and recommendations. We have organised our responses to each comment numerically below. In order to address the concerns raised by the reviewers, we have further refined both our manuscript and the associated software (LPhy and LPhyStudio).

### Reviewer 1

This paper presents a language for describing models and data in the context of statistical modeling of phylogenetic genetics. At present, the framework can be used for two purposes: - to simulate data and to setup input files for BEAST2. The authors indicate that they plan to extend the program to generate input files for other Bayesian phylogenetic frameworks in the future. The main content of the paper is to demonstrate how one would use the language to setup different models, by example. It also illustrates some of the features of an interactive program called LPhy Studio. I believe that the specification language, and, more importantly, the underlying engine that produces output based on the inputted specification, are value contributions to phylogenetics.

I assume that the code for generating data according to the different models already existed and so the main technical contribution here was probably to implement a parser for the specification language.

However, I do have some questions.

1. First, I think that the paper should say something about language features, e.g.: what language is the syntax modelled on, do variables have types?

All distributions and functions possess a return type, which can include an array of the given return type with vectorization. Consequently, variables have types, although these are not explicitly declared in the script. The same applies to arguments. Arguments must adhere to the type specified by the distribution or function, but arrays of the corresponding type are also permissible for vectorization. We have clarified this aspect in both the manuscript and the online language definition.

The syntax of this language shares similarities with JAGS and BUGS (in terms of random variable specification), Stan (with regards to data and model blocks), and R (concerning function vectorization and optional arguments). The syntax is elaborated further online and internally defined through an ANTLR grammar. We have now incorporated this information into the manuscript as well.

2. Are the methods overloaded?

Generally, the language accommodates argument overloading for distributions, provided that the type signatures differ (as is required in the underlying Java language implementation). Additionally, the language offers flexibility in allowing optional arguments, which can be considered another form of overloading. We have expounded on this in both the manuscript and the online resources.

3. When does the Studio flag errors, does it check syntax before you execute or only during execution?

It should be considered a scripting language, in the sense that syntax is checked during execution.

4. The examples use Greek letters. Is that required? The section on "Language Features" should contain more on this.

Greek letters are indeed supported but not mandatory. The language accommodates standard alphanumeric and Greek characters, as well as any Unicode characters. However, in LPhyStudio, support is more confined to standard ASCII and Greek characters for certain features, including (i) converting LaTeX-style parameter names to Unicode in the console, and (ii) converting model descriptions to LaTeX.

5. Why is this not a Python package? I believe that scripting is best done using a scripting language. I don't see why the parser and executor are written in Java. Even if there are good reasons for providing this in a Java framework, I suggest that providing a Python (or R?) package with the same functionality, as this would make this much more widely usable.

LinguaPhylo is a novel domain-specific model specification language. Its ultimate specification is based on an EBNF grammar (https://www.iso.org/standard/26153.html), and we internally utilise ANTLR to generate Java classes that parse this grammar into Java objects. Notably, ANTLR supports various target languages, including Python, C++, and JavaScript, making it relatively straightforward to generate a grammar parser for the LPhy grammar in other general programming languages. We have clarified this point in the manuscript.

LPhy is not a Python package since Python is a general programming language. Our intention was to create a domain-specific specification language, limiting LPhy to exclude flow control and other general language features to maintain simplicity. If LPhy were a package within a general programming language, programming aspects would likely seep in, making scripts less accessible to non-programmers.

6. The paper mentions a command line program for converting scripts into Beast2 configurations. There should also be a command line program for running a script to create simulated data, too.

We have added a command line program for simulation, and it is now described in the manuscript and on the website. Thank you for the suggestion.

7. I took a closer look at LPhy Studio and came across several issues. First, there is no installer, so one has to download a bunch of files and then run a jar from the command line. That is not state-of-the-art.

We have now included an installer with the latest LPhyStudio release available on GitHub.

8. Second, the program is hard to use because it does not contain the standard menus and menu items. In particular, there is no Edit menu, and therefore no cut, copy, paste, delete, undo and redo menu items.

We have added an Edit menu, and copy/paste/undo/redo items under the Edit menu.

9. The File menu doesn't contain a New... menu item and so program appears to only support one open document at a time. There is no print menu item.

We have added “New” and “Print” items to the Menu.

10. The File menu contains a Tutorials submenu that doesn't belong there.

The “Tutorials” submenu has been renamed to “Tutorial scripts”. This submenu contains scripts used on the website’s tutorial pages.

11. The interface itself is bare bones. There is no tool bar with the usual useful items. All content is organised into two tab panes and a text area, and the tab panes are not splittable, and so only a limited amount of content can be viewed together, even on a big screen.

We appreciate your feedback. We have now incorporated a toolbar featuring frequently used items (new, cut, copy, paste, undo, redo) into our software. We considered the possibility of implementing a split panel; however, we observed that the information in our panels requires a significant amount of vertical space (e.g., phylogenetic trees, substitution matrix). Displaying this information in a horizontally split panel would compress it excessively. As a result, we have opted to retain the current two-panel layout.

12. The model text editor appears to be a single line-editor. There does not appear to be any (easy) way to change something that has been entered (no undo redo, and no editing of the model script?).

We have now incorporated undo/redo keyboard shortcuts and added undo/redo options to the toolbar for the editor console. The model panel is designed to display previously executed commands, akin to the command history in a terminal. These historical commands are not intended to be editable; however, existing variables can be overwritten by subsequent commands that assign a different value or generator function to the variable.

13. The single-line editor doesn't have a history buffer.

Thank you for your helpful suggestions, we have now included a history buffer for the single-line editor console.

14. Looking into the code of LPhy Studio reveals that the UI is implemented using AWT and Swing. These technologies are severely outdated and explain why the UI of LPhy Studio feels so minimalistic and dated. However, the website mentions that start-of-the-art technologies are used?

The main emphasis of our paper lies in the LPhy model specification language, grammar parser, and modular software design, rather than GUI design. We have included a description of the language parser in the manuscript and online resources. Our software is built using contemporary development tools such as Java 17, ANTLR4, and Gradle v7, as well as a modular software extension mechanism utilizing the Java Service Provider Interface (SPI) and the Java Platform Module System (JPMS).
We agree that Swing, a Java-based graphical user interface (GUI) toolkit, can be considered somewhat outdated compared to newer GUI technologies like JavaFX or various web-based frameworks. However, Swing-based applications continue to be used and supported, and the API is mature and fully functional. Nevertheless a future goal for this project that is not within the scope of the current work is to develop a JavaFX or web-based UI.

15. The idea of representing phylogenetic analyses as graphs and generating a methods section ("narrative") from the graph to provide a precise, human readable description of the analysis, including citations, should be credited to Daniel Huson. This idea was presented by him in 2018 at a phylogenetic meeting in Dunedin. This presentation contained a figure showing a split window, with a graph representing a phylogenetic
    analysis above and a description of the analysis, together with all relevant citations below, very similar to Figure 2 of the manuscript. Alexei Drummond was present at the meeting and commented favourably on the idea at the time.

    The current wording of the manuscript might suggest that this is a new idea due to the authors and so it would be prudent to add an appropriate sentence to avoid that impression, something like this:

    "The idea of explicitly modelling the for provenance of a phylogenetic analysis as a graph, and using this graph to generate a textual description of the analysis, together with all relevant citations, was introduced in 2018 with version of 5 of SplitsTree (Huson and Bryant, 2006)."

We have revised the manuscript to emphasize that Probabilistic Graphical Models (PGMs) were initially introduced in Bayesian phylogenetics by Hohna et al. (2016). We note that the concept of generating textual descriptions for phylogenetic models was originally conceived by Alexei Drummond in 2006, during the development of the first version of Geneious. But in an academic context, we have included a statement acknowledging the development of automatic natural language descriptions of phylogenetic analyses in MEGA4 and SplitsTree5.

### Reviewer 2

The manuscript "LinguaPhylo..." attempts to solve the problem of simplifying descriptions of phylogenetic models to increase both repeatability and consistent description for methods. They do so by developing a simple model language, superficially similar to what one might find in other packages, that can theoretically stand independently of a particular software package, although currently the only integration is for BEAST 2.

The concept is straight-forward enough, although the practicality and complexity remains to be seen through actual use and, while adoption by other related software packages such as RevBayes or BEAST would likely be necessary for it to gain general traction.

In additional to the LPhy language, two tools are mentioned, an independent GUI, LPhyStudio which is designed to create graphical representations of models as well as "methods ready" descriptions of the model (not dissimilar to the "figure caption generation" seen in other packages such as MEGA), as well as LPhyBeast which is designed to create an Beast2 input XML from a LPhy model.

A few general issues strike me, none of them major.

16. The authors abbreviate "LinguaPhylo" as "LPhy" and state it is pronounced "el-fee" (p. 2). That is a losing battle as absolutely everyone is going to pronounce it "el-fai" given that it is "fai-logenetics" and not "fee-logenetics".

Thank you for your comment, we have adjusted the manuscript to point out that this is how we pronounce it, though we remain open to different pronunciations.

17. I am not an expert but the colors used in the figures, generated by LPhyStudio, including not just graphical representations but even color-coding of the LPhy text as shown throughout the manuscript, is very likely not color-blind friendly as it heavily makes use of green and red (reddish-appearing) colors (generally speaking, lime green (#00FF00) is not a particularly good color for text anyway as it is too bright on a white background)

Thank you for your suggestion. We have adjusted our software to use a colour-blind friendly palette for generating graphical representations and text highlighting.

18. I'm sure there is a logic to it, but I cannot quite make out within the LPhy language when a parameter is set with an = vs. a ~. I think it might be deterministic vs. stochastic, but the authors never specify this (if correct, I would suggest p. 3 where these types of generators are discussed).

The "=" symbol is utilised for assigning deterministic or constant values, while the "~" symbol is employed for specifying stochastic variables. We have incorporated a description of this usage in both the manuscript and the online resources (https://linguaphylo.github.io/features/).

19. The very first example of the language (Listing 1) is meant to be simple, but specifies the taxa "names" as the numbers 1 through 10. This inadvertently makes it appear that the language requires numbered taxa rather than named taxa, which I do not believe to be true.

The language supports standard alphanumeric, greek characters and numbered taxa names. We have added a clarification of this in the manuscript.

20. There is at least one typo, p. 3 "distributions"
21. While I know what it means, the authors use "i.i.d." at least twice without ever defining it. It should be defined at the time of first use

We have now clarified this in the manuscript, this term has now been defined before its usage.

22. The authors do not explain that the parameter "conc" is an abbreviation for "concentration" other than secondarily through the automatically generated natural text description toward the end of the manuscript on p. 10.

We have added a sentence explaining the “conc” abbreviation in the manuscript.

23. The natural text narrative in the examples have some oddities to them, but to be fair I'm not sure there is a way of fully auto-generating this sort of text in a perfect manner. Just as some examples, in the text shown on p. 10,

** The second sentence ends with "and a dataType" which makes no logical sense.

** The last two sentences begin with "The delta has a..." and "The epsilon has a..." when grammatical logic would suggest not starting either sentence with the word "The" or adding an additional explanation of what delta and epsilon refer to, e.g., "The delta of the error model..." and "The epsilon of the error model..." or something to that effect. I could nitpick other parts of the output, but as I said, I'm not sure it is possible to make a perfect one-stop solution to this natural text problem.

Thank you for your suggestions, we have improved the grammar in the automated text generation to take your comments into account. We have updated the example in the manuscript and online.

### Reviewer 3

This manuscript presents LinguaPhylo (LPhy), a language for easily defining models for BEAST 2 analysis. LPhy is much simpler than the normal (complicated) XML format, a welcome feature for BEAST 2 users. It is also coupled with LPhyStudio, a graphical interface, and LPhyBEAST, a command-line tool to convert the LPhy to the XML file.

24. Overall, these tools are certainly worthwhile to the BEAST community. However, I failed to understand how useful it is to other Bayesian software or phylogenetic software, in general. If LPhy is presented as a “language” (as the title and abstract suggest), then there should be information how it can be generally adopted, outside of BEAST. This is currently missing.

The LPhy scripting language is designed for specifying phylogenetic models. Our paper demonstrates its application for BEAST2, which enables the generation of simulated data and executable BEAST2 XMLs. We anticipate that the LPhy language will soon be expanded to accommodate other Bayesian software. For instance, RevBayes support could be achieved by implementing a parser that translates LPhy model specifications into functional RevBayes scripts.

25. For phylogenomic inference, mixture models are now widely used, especially quite important to address deep or controversial phylogenetic questions; and also useful for more recent questions such as incomplete lineage sorting. I don’t find any information how to specify mixture models. (e.g, can I use the CAT model in PhyloBayes?)

It is already possible to implement simple phylogenetic mixture models in LPhy (e.g. by using the vectorisation support to generate alignment columns from different substitutions models along the same phylogenetic tree). However DirichletProcess prior models like CAT are not currently available in LPhy. We plan to implement the DirichletProcess prior in version 1.5 of the software (<https://github.com/LinguaPhylo/linguaPhylo/issues/329>), but that is outside the scope of the current piece of work.

26. Is LPhy format case-sensitive?

Yes, LPhy is case-sensitive. We have added a clarification for this online (https://linguaphylo.github.io/features/) and in the manuscript.

27. And is it possible to adopt LPhy for maximum likelihood analysis? This can be seen as a subset of the current language (i.e., excluding the priors). So I don’t see why not. One can even think about a more lightweight version of LPhy, which will benefit the ML community.

LPhy focuses solely on defining probabilistic models, rather than dictating the mode of inference for the model. Various methods can be employed for inference under the model, such as Maximum a posteriori, MCMC-based posterior sampling, or Maximum likelihood based on the arguments of the PhyloCTMC distribution (phylogenetic likelihood).

As a model definition language, LPhy does not prescribe any specific inference procedures. Downstream tools, like LPhyBEAST, establish the inference processes. It is entirely possible to create a downstream tool that implements Maximum Likelihood inference or to develop adapters for popular ML packages like RaxML and IQTree.
